# Recalcitrant lupus miliaris disseminatus faciei improved by cyclosporine monotherapy

**DOI:** 10.1016/j.jdcr.2022.06.038

**Published:** 2022-07-06

**Authors:** Hyeok-Jin Kwon, Kyung-Deok Park, Dong-Wha Yoo, Jeong-Wan Seo, Ki-Ho Kim, Jung-Ho Yoon

**Affiliations:** Department of Dermatology, College of Medicine, Dong-A University, Busan, Republic of Korea

**Keywords:** cyclosporine, interleukin 2, lupus miliaris disseminatus faciei, type 1 immunity, GA, granulomatous rosacea, IL, interleukin, LMDF, lupus miliaris disseminatus faciei, Th1, T helper 1

## Introduction

Lupus miliaris disseminatus faciei (LMDF) is a chronic cutaneous granulomatous disease characterized by multiple smooth reddish or yellowish papules on the central face, especially the lower eyelids and perioral area.^1^, ^2^, ^3^ Owing to its clinical manifestations, LMDF has been considered as a variant of granulomatous rosacea (GA).^1^^,^^2^ However, LMDF may be a distinct entity, different from GA, sarcoidosis, and cutaneous tuberculosis.^1^^,^^2^

Treatment of LMDF is usually challenging.^1^^,^^3^ Tetracycline, isotretinoin, hydroxychloroquine, dapsone, systemic steroids, pulsed dye laser, topical steroids, and topical calcineurin inhibitors are frequently used to treat LMDF; however, their therapeutic efficacy remains controversial.^1^^,^^3^^,^^4^

Herein, we report a recalcitrant case of LMDF that was resistant to minocycline, isotretinoin, and systemic steroid plus dapsone with topical tacrolimus but dramatically improved with cyclosporine monotherapy. Interestingly, cyclosporine has rarely been introduced as an LMDF therapeutic regimen.^5^ Therefore, we also discuss the therapeutic efficacy of cyclosporine in this case, considering the immunopathogenesis of granuloma formation.

## Case report

**Fig 1:**
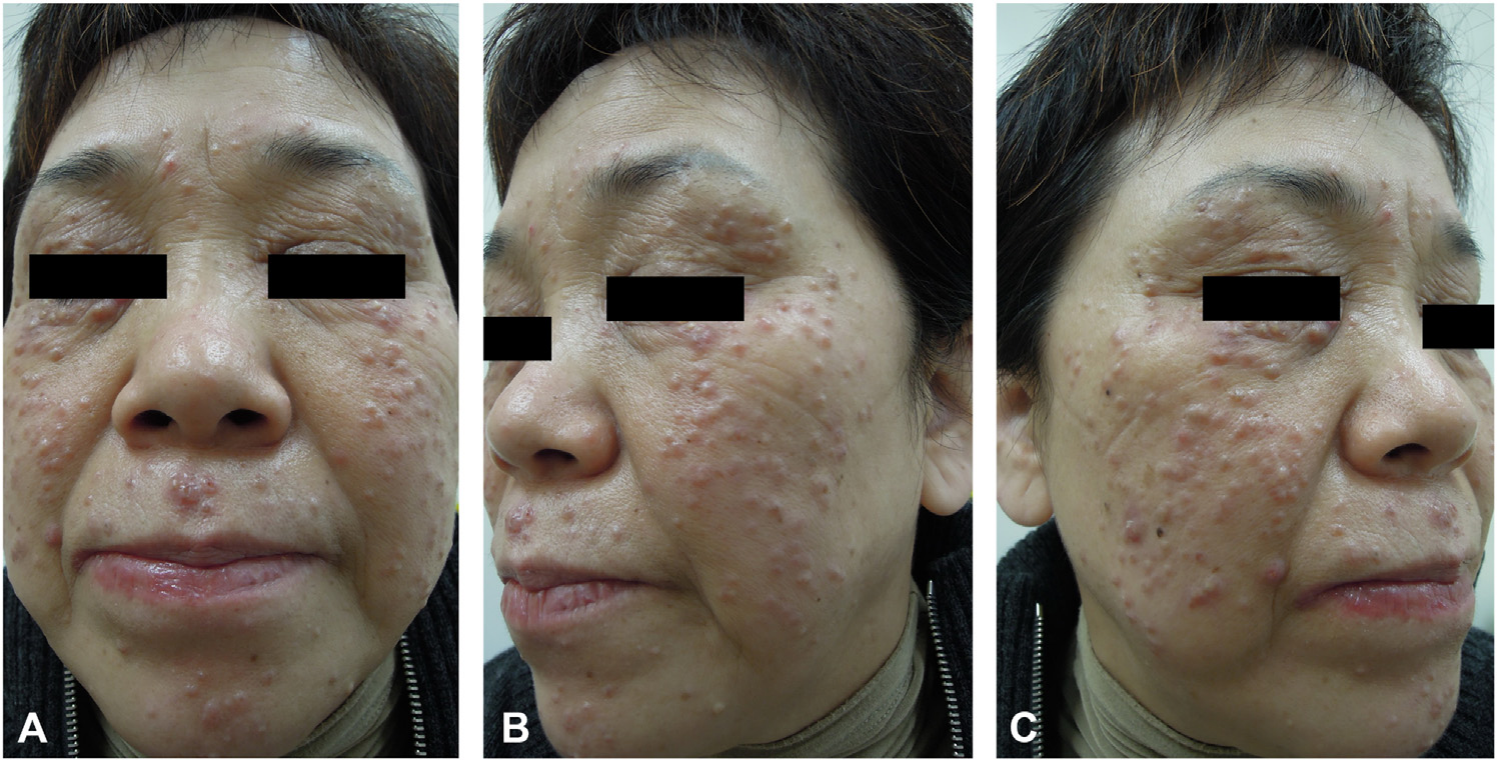
Cutaneous findings at the patient’s first visit. (**A-C**) Multiple erythematous and *yellowish* papules on the patient’s entire face.

**Fig 2:**
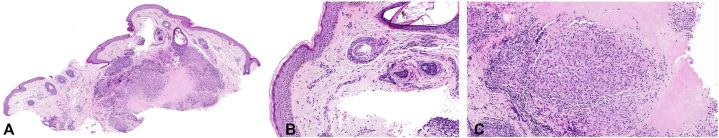
Histopathological features of the cutaneous lesions on the face. **A,** Perifollicular granulomatous change with central caseating necrosis is prominent, and peripheral inflammatory infiltration is observed. Hematoxylin and eosin staining, original magnification × 10. **B,** Focal hyperkeratosis and pigmentary incontinence are found. Hematoxylin and eosin staining, original magnification × 20. **C,** On higher magnification, epithelioid granuloma, caseating necrosis, and peripheral lymphocyte infiltration are identified. Hematoxylin and eosin staining, original magnification × 50.

A 60-year-old Korean woman visited our department because of multiple erythematous and yellowish papules on her entire face that lasted 6 months. She had no subjective symptoms or medical history. She stated that the cutaneous lesions had started on both lower eyelids and the perioral area and had spread throughout the face within 1 month (Fig 1, *A*–*C*). Clinically suspecting LMDF, GA, and sarcoidosis, we initially performed a skin biopsy of the lesions. At a lower magnification, perifollicular granulomatous changes were prominent, with peripheral inflammatory infiltration and central caseating necrosis (Fig 2, *A*). Additionally, focal hyperkeratosis and pigmentary incontinence were observed in the epidermis (Fig 2, *B*). At a higher magnification, epithelioid granulomas with peripheral lymphocytic infiltration were observed (Fig 2, *C*). On immunohistochemistry, both acid-fast and periodic acid-Schiff staining results were negative. Tissue cultures for tuberculosis were also negative. Further, we performed blood tests, including those for serum angiotensin-converting enzyme, erythrocyte sediment rate, and serum calcium, interferon gamma release assay, and chest X-ray; however, no noticeable findings were observed. Based on these clinical and pathological findings, the patient was diagnosed with LMDF.

**Fig 3:**
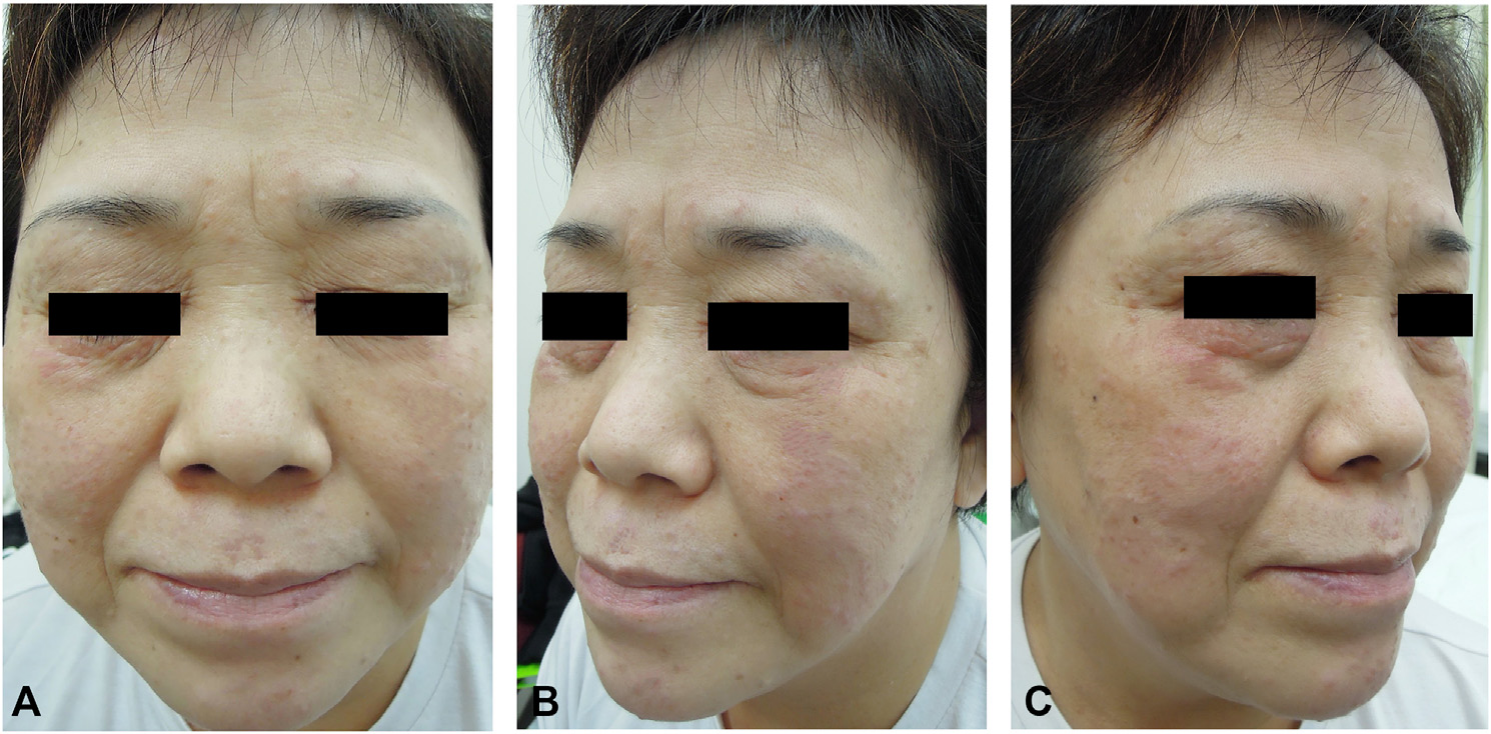
Improved cutaneous lesions on the patient’s face after 1 month of cyclosporine monotherapy. **A-C,** Dramatic reduction in erythematous and *yellowish* papules on the patient’s face during cyclosporine therapy.

We first treated the patient with oral minocycline (100 mg twice daily) and isotretinoin (20 mg twice daily) for 3 weeks each; however, there was no improvement during these 6 weeks of treatment. Therefore, we changed the therapeutic regimen to oral prednisolone (10 mg twice daily) plus dapsone (100 mg once daily) with topical 0.03% tacrolimus for 1 month. Despite these combination therapies, we did not notice any treatment effect. Furthermore, the patient complained of a burning sensation after using topical tacrolimus. Consequently, we stopped these regimens and prescribed oral cyclosporine (50 mg twice daily) monotherapy for 1 month. During the treatment period, the erythematous and yellowish papules on her face disappeared significantly (Fig 3, *A*–*C*). After 1 month, she was satisfied with the treatment outcomes but did not want to take cyclosporine any longer because of nausea. Hence, although we explained that a longer treatment period would be needed, we discontinued cyclosporine therapy, and her follow-up was lost.

## Discussion

LMDF is comparable to GA; however, it has distinctive clinical and pathologic features.^1^^,^^2^ LMDF usually presents as asymptomatic flesh-colored or mild erythematous papules without an erythematous base, whereas GA typically has an erythematous base with vascular symptoms, such as flushing, burning, or itching.^1^^,^^2^ Histologically, it is characterized by epithelioid granulomas with central caseating necrosis.^1^^,^^2^

Despite its obscure etiology and pathogenesis, there exists a hypothesis that an immune response to pilosebaceous units and the resulting antigen release into the dermis is a possible mechanism promoting granuloma formation in LMDF.^1^^,^^3^ The immunopathogenesis of granuloma formation relies on type 1 immunity in which T helper 1 (Th1) cells play a significant role in the production of interleukin (IL)-2 and interferon-γ, and subsequent T cell proliferation and macrophage activation induce cell-mediated immunity.^6^, ^7^, ^8^ Macrophages are essential for the creation of granulomas by engulfing causative antigens and presenting them to CD4^+^ helper T cells, which sequentially make these cells differentiate into Th1 subtypes.^8^ Consequently, activated Th1 cells accelerate macrophage functions and vice versa.^6^, ^7^, ^8^ Besides, macrophages involved in granuloma formation have an “epithelioid” shape easily found in the pathologic features of numerous granulomas, including LMDF (Fig 2, *C*).^1^^,^^2^^,^^8^

There are many therapeutic options for LMDF, such as tetracycline, isotretinoin, hydroxychloroquine, dapsone, systemic steroids, pulsed dye laser, topical steroids, and topical calcineurin inhibitors; however, the efficacy of these treatments remains debatable, and there exists no formulaic therapeutic guideline.^1^^,^^3^^,^^4^ In our case, we applied systemic steroids, dapsone, and topical calcineurin inhibitor in combination to the patient because these agents have been shown to be relatively more effective than others.^3^ Nonetheless, no meaningful improvement was observed; therefore, we changed the therapeutic regimen to cyclosporine monotherapy, considering the immunopathogenesis of granuloma formation described above.

Cyclosporine is an immunomodulatory agent that affects T lymphocytes by binding to cyclophilins and consequently inhibiting the transcription of the IL-2 gene.^9^ We speculated that cyclosporine showed its therapeutic efficacy against LMDF by suppressing cell-mediated immunity by blocking IL-2 functions.^6^, ^7^, ^8^, ^9^ Meanwhile, Spadino et al^10^ reported 4 cases of disseminated granuloma annulare successfully treated with cyclosporine, and Sardana et al^5^ reported its satisfactory medicinal effect in 1 LMDF case. Consequently, we suggest that type 1 immunity is part of the entire pathophysiology of LMDF and blocking type 1 immunity can be a possible therapeutic target in terms of the immunomodulatory effect of cyclosporine on Th1 cells.^6^, ^7^, ^8^, ^9^ However, we could not definitely exclude the possibility of spontaneous resolution.^1^^,^^2^

In conclusion, we report that cyclosporine may be an effective treatment option for LMDF. To the best of our knowledge, cyclosporine has rarely been reported as a therapeutic agent in LMDF.^5^ Hence, our case additionally shows the possibility of using cyclosporine in the treatment of LMDF. Moreover, type 1 immunity and IL-2 could play a significant role in the immunopathogenesis of granuloma formation in LMDF. Therefore, we propose that not only clinical studies using cyclosporine as a primary treatment option but also translational research that can support the pathogenic role of type 1 immunity in LMDF are needed.

## Conflicts of interest

None disclosed.
